# Collagen composition in equine exuberant granulation tissue reflects tissue immaturity

**DOI:** 10.1371/journal.pone.0335179

**Published:** 2025-11-06

**Authors:** Lena Partusch, Catrin Sian Rutland, Ann Martens, Charis Du Cheyne, Ward De Spiegelaere, Jule Kristin Michler

**Affiliations:** 1 Department of Morphology, Faculty of Veterinary Medicine, Ghent University, Merelbeke, Belgium; 2 School of Veterinary Medicine and Science, Sutton Bonington Campus, Faculty of Medicine and Health Sciences, University of Nottingham, Nottingham, United Kingdom; 3 Department of Large Animal Surgery, Anaesthesia and Orthopaedics, Faculty of Veterinary Medicine, Ghent University, Merelbeke, Belgium; 4 Physiology and Pathophysiology, Dpt. of Biological Sciences and Pathobiology, University of Veterinary Medicine Vienna, Vienna, Austria; 5 Institute of Anatomy, Histology and Embryology, Faculty of Veterinary Medicine, Leipzig University, Leipzig, Germany; GH Raisoni College of Engineering and Management Pune, INDIA

## Abstract

Exuberant granulation tissue (EGT) is a second intention wound healing disorder. It commonly occurs in the distal limb of horses. EGT causes significant increase in the duration and cost of treatment, potentially leading to the decision not to pursue treatment and euthanize the patient. The underlying pathomechanisms of this fibroproliferative disorder remain unclear, particularly in terms of collagen composition and the association between myofibroblasts and blood vessels. This study investigated the collagen composition in naturally occurring EGT following trimming in 19 horses (EGT group). In both the superficial and deep wound beds of EGT-affected horses, the collagen distribution was assessed and compared to control wounds (n = 6 horses, control group, punch biopsies) using histology. Immunofluorescence was performed to colocalize activated alpha smooth muscle actin-positive myofibroblasts in EGT as well as angiogenic markers. Our histological findings showed significantly higher amounts of immature collagen (type III) in the superficial and deep regions of EGT compared to the controls while the total amount of collagen in both groups did not differ significantly. In EGT, occluded microvessels and endothelial cell hypertrophy were present in the deep layer and myofibroblasts were ubiquitously found in the whole wound bed. Markers for intermediate filaments were reduced in the superficial region. In conclusion, collagen composition in EGT differed significantly from control wounds, indicating tissue immaturity. Consequently, promoting tissue maturation towards a more mature ECM composition could serve as a valuable target for future therapeutic interventions enabling better regeneration.

## Introduction

In horses, cutaneous wounds are the second most common cause of death according to previous studies and reviews [1–3]. Wounds at or below the level of the carpus or tarsus are often heavily contaminated. These injuries frequently involve not only the skin but also underlying structures such as tendons and bone. As a result, they typically heal by second intention. However, second intention healing in this region is challenging and often leads to the development of excessive, almost tumor-like growth of granulation tissue—also known as proud flesh, hypergranulation, or exuberant granulation tissue (EGT) [4–8].

The complications related to EGT development result in prolonged treatments, increased costs and up to 25% of equine patients eventually suffer from decreased performance, early retirement or are even euthanized [3]. The exact mechanism causing the hyperproliferation of granulation tissue is still unknown. Knottenbelt et al. listed 12 factors delaying wound healing, including local and genetic conditions [9]. Further, other several contributing factors to EGT formation such as higher movement, increased skin tension and the higher risk of contamination at the distal limb are considered as potential triggers [10–12].

Equine wound healing mechanisms in distal limbs primarily rely on epithelialization and not contraction [13]. This is also the case in human wound healing. Some authors suggest that EGT bears some similarities to human keloid scars as they are both fibroproliferative disorders with a comparable degree of dermal fibrosis [14]. However, in contrast to human keloids, equine EGT lacks an epithelial cover and is chronically infected.

Microscopically, EGT classically divides into a superficial inflammatory layer and a deep fibrotic layer [15]. Studies on the inflammatory aspects of EGT indicate that hypergranulating wounds are stuck in the proliferative phase of the wound healing cascade, preventing the tissue from both growing an epithelial cover and from entering the final remodelling phase [6,15,16]. Additionally, coordination between the myofibroblasts appears to be impeded, leading to an overproduction of extracellular matrix (ECM), lack of wound contraction, and the inability of keratinocytes to epithelialise the wound surface [17,18].

Therefore, the first aim of our study was to analyze the ECM components, in particular collagen type I and type III distribution, within EGT. These two collagen types were selected as they represent the most functionally relevant collagens in dermal wound healing—type III is typically associated with early granulation tissue, while type I dominates in the remodelling phase and contributes to tissue strength when the wound advances to scarring [19]. The collagen types were semi-quantified and the ratios of immature versus mature fibres in EGT were compared to control wounds using histology, e.g., picrosirius red stain. Additionally, qualitative collagen detection via immunofluorescence was performed in EGT.

The imbalance of excessive granulation tissue growth with overproduction of ECM components and aberrant blood vessel formation is a hallmark feature in EGT formation. Histologically, there is a significantly higher number of occluded microvessels and endothelial cell hypertrophy in equine distal limb wounds [20]. Using spectroscopy, Celeste et al. reported hypoxic conditions during the inflammatory phase of wound healing next to a reduced cutaneous wound temperature in limb wounds developing EGT [21,22]. In general, hypoxic conditions induce a variety of pro-angiogenic pathways [23]. To deliver a comprehensive picture, the second aim of the present study was to link our ECM results to angiogenic marker expression to visualize the morphology of the newly formed vessels.

The third aim of our study was to precisely describe the cells that so abundantly produce the ECM in EGT. *In vitro*, isolated equine dermal fibroblasts, from the body and limbs, have both previously shown hypoxia-induced factor 1A-related upregulation of ECM production [24], which is in accordance with human studies showing mRNA upregulation of procollagen genes in hypoxic conditions [25]. Besides ECM production, other key features of myofibroblasts in wound healing are the upregulation of Alpha smooth muscle actin (α-SMA), mobility in the wound bed, and intermediate filament vimentin expression as a lineage marker of all mesenchymal descendants. Theoret et al. histologically examined and reported the absence of myofibroblasts in human keloids, also a second intention wound healing disorder with hyperproliferation, and compared it to EGT [14]. Based on their findings, we used the named antibodies (anti-α-SMA-antibody for myofibroblast detection and anti-vimentin-antibody for detection of mesenchymal cells) and performed immunofluorescent colocalization assays enabling multiple staining targets in each EGT section. Vimentin and α-SMA expression were detected and linked to their colocalization with collagen.

## Materials and methods

Exact information on supplier and catalog numbers of chemicals and consumables is supplied in S1 Table.

### Animals and sample material

EGT from 19 horses ($\bar{\text{x}}$ = 4 years, range: 1–14 years) was harvested following trimming procedures (for detailed data on the horses, please see S2 Table). The horses were presented at the Equine Clinics of Ghent University for the treatment of their hypergranulating wounds. The tissue was obtained as surgical waste material and therefore required no ethical approval from the Ghent University ethical committee. The tissue pieces were transferred to the laboratory in 50 ml falcon tubes, submerged in PBS, and then cut into processable pieces with a surface of 1 cm^2^. The trimmed EGT tissue pieces were then formalin-fixed for 24 h and subsequently paraffin-embedded (FFPE) using a tissue processor (STP120 Spin, Thermo Fisher Scientific, USA) and a tissue embedding station (Microm, EC350, Thermo Fisher Scientific, USA). The orientation of the tissue pieces enabled sections to be cut perpendicular to the wound surface.

To compare collagen distribution and to undertake quantification in EGT, a control group was used for comparison. FFPE granulation tissue samples were obtained from six horses (12 yrs ± 5 years) with experimentally induced, non-contaminated wounds from a study previously published [26]. The wounds were previously created in a dorsomedial direction in both metacarpi during aseptic surgery. These control wounds macroscopically did not develop EGT at any time during the wound healing process. Two weeks after the wounding, and on day 19, punch biopsies (6 mm granulation tissue and 2 mm skin of wound edge) were performed and the wound tissue was fixed using 10% neutral buffered formalin and processed [26,27].

#### Ethical statement.

The control samples utilized in this study were originally collected as part of a previous study conducted at Ghent University, which received approval from the Ethical Committee of the Faculties of Veterinary Medicine and Biological Engineering on 3 February 2014 (approval number 2014/183). No additional ethical approval was required for the current study, as the tissue samples were considered surgical waste generated during routine treatment procedures. All experiments were performed in accordance with relevant institutional guidelines and regulations. As tissue from a previous study was utilized, this additionally conformed to the 4Rs (Reduction, Refinement, Replacement, Responsibility).

### Histological staining

For Masson’s trichrome, Herovici and picrosirius red staining, 7 µm sections were deparaffinized and stained using the subsequent protocols.

#### Masson’s trichrome staining.

The sections (n_EGT_ = 17 horses; n_control_ = 3 horses) were incubated in Bouin’s fixative for 60 min and then rinsed in distilled water, followed by a 10 min incubation in hematoxylin solution. The slides were then washed for 5 min under running tap water and immersed three times in Biebrich scarlet-acid fuchsin solution. After washing in distilled water, the sections were incubated in phosphomolybdic-phosphotungistic acid solution for 20 min and subsequently immersed in aniline blue solution for 8 min. The sections were then rinsed in distilled water, followed by an 8 min incubation in 1% acetic acid solution.

#### Herovici staining.

A Herovici staining solution consisting of 50 ml van Gieson’s solution, 50 ml of 0.05% methyl blue (diluted in distilled water), 10 ml glycerol and 0.5 ml lithium carbonate (sat. aqu.) was prepared. The sections (n_EGT_ = 19 horses; n_control_ = 6 horses) were incubated in hematoxylin for 10 min, followed by 5 min washing under running tap water. Subsequently, the sections were immersed in the Herovici staining solution for 2 min and directly washed in 1% acetic acid solution for 2 min.

#### Picrosirius red.

The sections (n_EGT_ = 19 horses; n_control_ = 6 horses) were stained using a standard staining kit (cat. no. ab150681, Abcam, UK). Briefly, sections were incubated 60 min at RT in the picrosirius red staining solution and directly washed twice in the provided acetic acid solution for 1 s each. Subsequently, the sections were dipped in absolute ethanol, dehydrated in an ascending alcohol series and mounted using DPX. Image acquisition was performed using an upright Olympus BX61 microscope and CellSens software (Olympus, Japan) combined with an Olympus linear polarisator in a dark environment. The slides were fully scanned with a 20X objective with a constant exposure time (22.73 ms). Image analysis was performed using the pixel classifier in QuPath (version 0.4.4) [28]. The classifier was trained on picrosirius red stains of control wounds categorizing red-orange pixels as thick fibres (collagen I), green-yellow pixels as thin fibres (collagen III) and black as background. Regions of interest (ROI) of at least 1 mm^2^ were randomly selected in both the superficial and deep layer. For each ROI, the ratio of thin versus thick fibres was calculated. In total, 57 superficial and 50 deep ROIs in EGT sections of 19 horses and compared to 17 superficial and deep ROIs in control sections of 6 horses. For each horse and layer, the mean ratio was built. The statistical analysis was performed by JMP (version 18.2.0) using a non-parametric Kruskal-Wallis-Test comparing the ratios of superficial and deep granulation tissue of EGT and control horses. A p-value less than α = 0.05 was considered statistically significant. In addition, the superficial layer depths were measured using three systematic jmp sampling measurements (located left, middle and right of each section) from all EGT and control horses, with a mean and standard deviation calculated and independent T-test conducted.

### Immunolabelling

#### Immunofluorescence.

Tissue blocks were cut (5 µm thick sections) using a microtome (HM355S, Thermo Fisher Scientific, USA), mounted on silanized slides, and deparaffinized in xylene, followed by a descending alcohol series for rehydration. For antigen retrieval, the slides were processed for 15 min in citrate buffer pH 6.0 diluted in distilled water in a steamer, then washed thrice for 3 min in PBS. Blocking and permeabilization was performed using 10% donkey serum in 0.1% Triton X 100 in PBS for 20 min at RT and then two washes in PBS.

The primary antibodies were prepared and applied as described in Table 1. The secondary antibodies were all diluted 1:500 in PBS and applied for 45 min at RT. Every antibody incubation step was followed by three washes in PBS. Immunofluorescent stainings were performed as double, triple or quadruple stainings.

**Table 1:** Primary antibodies used for immunofluorescent (IF) and immunohistochemical staining (IHC).

Antigen	Species	Supplier	Catalogue number	LOT number	IF/ IHC	Working dilutions (PBS) and incubation time
Alpha smooth muscle actin (α-SMA)	Mouse (monoclonal) Clone 1A4	Sigma Aldrich, now Merck Millipore	A2547	n.a.	IF	1:200; 90 min at RT
CD31	Rabbit (monoclonal)	Abcam	ab134168	GR177389−17	IF	1:50; overnight at 4°C
Collagen type I	Rabbit (monoclonal)	Abcam	ab138492	1000862−6	IF	1:200; 90 min at RT
Collagen type III	Rabbit (polyclonal)	Abcam	ab7778	1034103−1	IF	1:50; overnight at 4°C
					IHC	1:500; 120 min at RT
PDGFRα and PDGFRβ	Rabbit (monoclonal)	Abcam	ab32570	GR3241180−25	IF	1:200; 90 min at RT
					IHC	1:500; 120 min at RT
Vimentin (coupled to Cy3)	Mouse (monoclonal) Clone V9	Sigma Aldrich, now Merck Millipore	C9080	n.a.	IF	1:500; 90 min at RT

For double immunofluorescence, the sections were incubated with the primary antibodies against CD31, collagen type I, collagen type III or platelet-derived growth factor receptor (PDGFR). Subsequently, the secondary antibody Alexa Fluor® 488 Donkey Anti-Rabbit was applied followed by 15 min incubation with Hoechst®33342 (1:1000 diluted in PBS) for nuclear counterstaining. For triple staining, the sections were additionally incubated with the antibody for vimentin conjugated to Cy3. For quadruple staining, the first antibody was coupled with secondary antibody Alexa Fluor® 647 Donkey Anti-Rabbit. Next, the anti-α-SMA-antibody, followed by secondary antibody Alexa Fluor® 488 Donkey Anti-Mouse was applied. The anti-vimentin-antibody was incubated afterwards. and the nuclear counterstaining was performed as described previously. All slides were finally washed twice in PBS and mounted using Fluoroshield. Image acquisition was carried out using a Nikon TE2000 microscope (Nikon Europe B.V., Germany). As positive controls, equine FFPE skin sections were stained using the same protocol (S1 Fig). For negative control (unspecific binding of the secondary antibody), the primary was omitted.

#### Immunohistochemistry.

To substantiate the IF staining, IHC for PDGFR and collagen type III with DAB as chromogen was performed in samples from six horses. Sections were prepared as described previously. Antigen retrieval was performed using a microwave (150 s maximum power and 10 min 160W) in citrate buffer pH 6.0 diluted in distilled water. The sections were blocked for 30 min in 30% goat serum. The primary antibodies were prepared and applied as described in Table 1. Endogenous peroxidase was blocked for 5 minutes using 3% H_2_O_2_ in methanol. Subsequently, the EnVision+ System-HRP Labelled Polymer Anti-Rabbit was applied according to manufacturer‘s instructions, followed by DAB-visualisation with Liquid DAB+ Substrate Chromogen System. Hematoxylin staining was used as a nuclear counterstain, then the tissue was rehydrated and coverslips mounted with DPX. Image acquisition was conducted using an upright Olympus BX61 microscope and CellSens software (Olympus, Japan). Equine FFPE skin sections were stained using the same protocol as positive control (S2 Fig). Negative controls (secondary antibody control) were performed as described above.

## Results

### Histological and statistical analyses reveal different collagen composition in EGT

**Fig 1:**
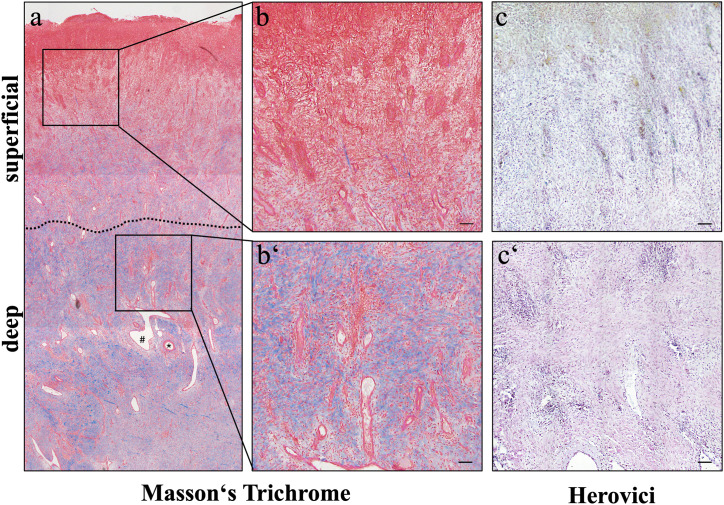
Masson’s trichrome and Herovici staining reveal distinct layering in EGT. Microscopically, EGT showed two clearly distinguishable layers when stained with Masson’s trichrome staining **(a)**. The superficial layer contained a high number of inflammatory cells (b, inset of **a)**, discriminated by their pink cytoplasmic staining. In the deep layer, an increased amount of blue stained collagen fibres was observed following Masson’s trichrome staining (b‘, inset of **a)**. The deep layer contained extensively branching blood vessels (* = artery; # = vein).

Masson’s trichrome staining was employed as a histological standard stain to morphologically evaluate the samples (Fig 1a, 1b). Collagen fibres were depicted in blue, revealing the total collagen amount, whereas a distinction of collagen types was not possible using this stain. In the superficial layer of EGT, Masson’s trichrome stained a high number of pink nuclei, indicating the inflammatory nature of the layer, which was predominantly infiltrated by immune cells. In the deep layer, the amount of blue stained collagen fibres increased. In contrast to Masson’s trichrome, the Herovici stain (Fig 1c) enabled the differentiation between immature (blue) and mature collagen (red to pink), allowing for the identification of the distinct fibre types. The Herovici staining showed immature collagen fibres (purple blue) in the superficial regions. The ECM fibres in the deeper regions were stained pink indicating mature collagen. Quantification of collagen distribution and composition was not undertaken, as the pixel classification of purple-blue versus pink proved not precise enough during our pre-experiments to yield reliable results.

With the Herovici staining, reticulin and immature fibres within the superficial layer were displayed in purple to blue (c). Additionally, nuclei were stained in black and cytoplasm in yellow, confirming the cell-rich superficial region. The mature collagen fibres within the deep layer were stained a more red-violet colour (c‘). All scale bars = 100 µm.

Picrosirius red staining of EGT and control wounds was used as a third technique to identify collagen, type distinction was performed using polarized light. Analysis of the stained sections (S3 Fig) revealed that the superficial layer of EGT never extended deeper than 1.5 mm from the wound surface ($\bar{\text{x}}$ = 1.066 mm; σ = 0.429 mm, n_EGT _= 19 EGT), which was significantly higher (p = 0.0028) in the control group ($\bar{\text{x}}$ = 1.4756 mm; σ = 0.152 mm, n_control _= 6).

**Fig 2:**
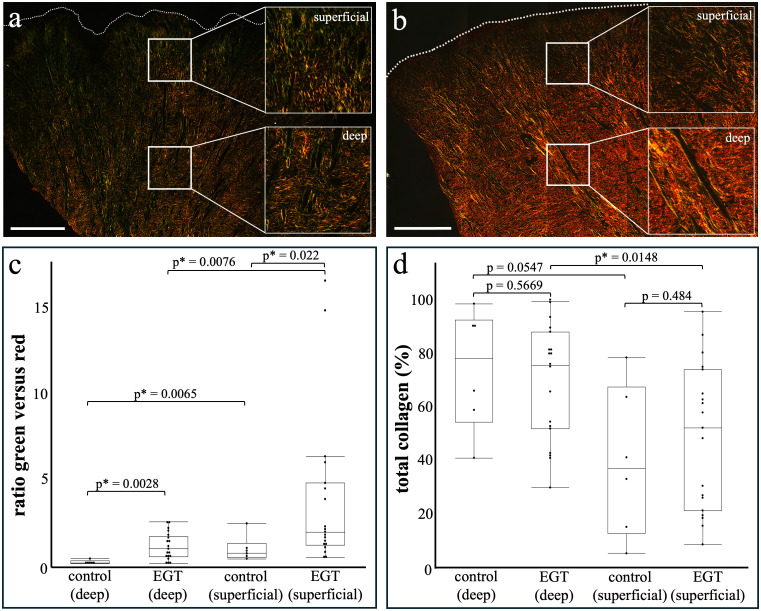
Significant differences in collagen composition of equine wounds based on picrosirius red staining. The superficial ROIs in EGT showed a significantly higher ratio of thin (green, collagen III) versus thick (red, collagen I) fibres than the deep layer **(c)**. Comparing both layers of EGT to the control group, a significant difference was observed with more thin fibres in EGT in both the superficial and deep ROIs of EGT.

EGT showed mainly thin fibres (stained green-yellow, immature collagen III) in the superficial region and a higher amount of thick fibres (orange-red, mature collagen I) in the deep layer (Fig 2a). Superficially, the thin fibres formed a network which was focally supported by thick fibres orientated perpendicularly to the wound surface, that were associated with the walls of newly formed blood vessels. In the deep layer of EGT, a higher fibre density was observed compared to the superficial layer. The fibre orientation in the deep layer was grid-like with a low interfibre distance. The sections from the control group showed a comparable architecture within the wound bed with a similar tendency towards more intensely stained and tighter packed fibres in the deep region (Fig 2b). In contrast to the EGT group, more thick fibres were observed in the granulation tissue within the control sections. These qualitative observations were supported by the statistical analysis: The superficial region showed a significantly higher ratio of thin versus thick fibres in both the EGT (p = 0.0076) and control group (p = 0.0065, Fig 2c) compared to the deep region. When the two groups were compared, the superficial region of EGT samples showed a significantly higher ratio of thin versus thick fibres compared to the control samples (p = 0.022). The same trend was seen in the deep layer, where the EGT samples had a significantly higher amount of thin fibres compared to the control group (p = 0.0028, Fig 2c).

Regarding the total amount of collagen (Fig 2d), the EGT wounds showed a significantly lower amount of collagen fibres in the superficial region compared to the deep layer (p = 0.0148). In the control wounds, the superficial and deep layers showed no significant differences in their total collagen amounts (p = 0.0547). No differences were seen when comparing the total collagen amount in the superficial (p = 0.484) and the deep (p = 0.5669) layers of the EGT and non-EGT wounds (Fig 2d).

Regarding the total amount of collagen (d), a significantly lower amount of collagen was seen in the superficial layer of the EGT compared to the deeper layer, this difference was not observed in the control group. Overall, there was no significant difference in the total collagen amount when EGT was compared to the control horses. All scale bars = 1 mm.

### Immunofluorescent staining of collagen in EGT confirms findings of picrosirius red staining

**Fig 3:**
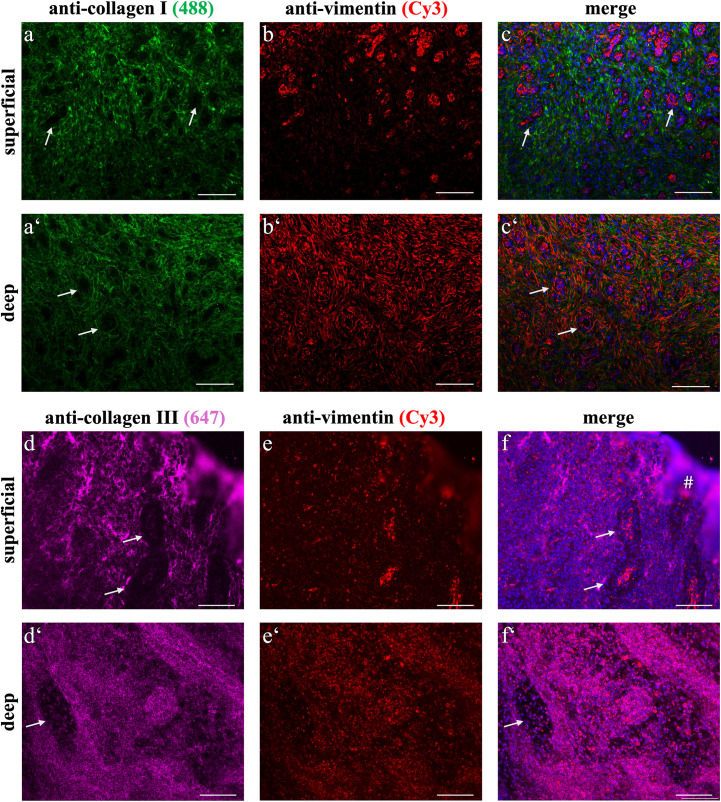
Immunofluorescent expression of collagen types in EGT. In the superficial, as well as in the deep layer, collagen type I immunofluorescence showed a network of cobweb-like fibres around the blood vessels (a, a‘). Vimentin was labelled positive in both layers, with an observed increase in the deep region (b, b‘, c, c’ represent merged staining images). The staining for collagen type III was present in every region of EGT (d, d‘) and no clear distribution pattern was observed. Vimentin (e, e’) was also observed in the collagen type III stained sections. f, f’ represent merged images. Blurred purple regions in d and f (#) were staining artefacts. Arrow = blood vessel. All scale bars = 100 µm.

The immunolabelling for collagen type I in EGT supported the histological findings with positive staining predominantly in the deeper wound bed (Fig 3). The superficial region in the EGT group showed less intense positive staining compared to the deep layer and a noticeable labelling of the vascular walls, which was underlined by vimentin co-staining (Fig 3a-3c). The most superficial myofibroblasts showed weak intracellular labelling for collagen type I. In the deep regions, the majority of the staining was observed in the ECM which labelled positively for collagen type I and exhibited a grid-like architecture.

The immunofluorescent staining for collagen type III (Fig 3d-3f) showed positive signal in the superficial as well as the deep region of EGT. Labelling was seen intra- and extracellularly, whereby the extracellular signal was observed to be cobweb-like, consisting of thin fibres that did not exhibit a clear distribution pattern or accumulation. Intracytoplasmic signalling showed perinuclear accumulation of collagen type III positive staining. These findings were derived from the positive staining observed in these slides. However, it is important to note some variability in immunofluorescent labelling as some slides did not produce positive staining. In a subset of samples, immunohistochemistry using DAB-labelling proved to be superior with regards to staining consistency (S4 Fig).

### Association of myofibroblasts and blood vessels in EGT α-SMA and vimentin expression in EGT: Layer-specific differences

**Fig 4:**
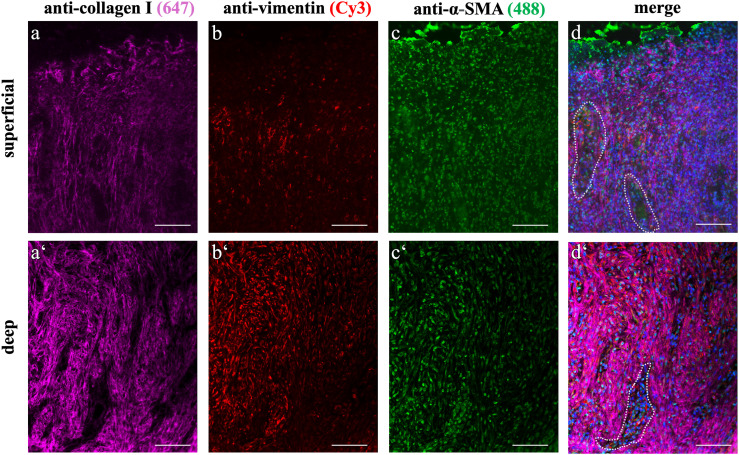
Immunofluorescent overview of EGT: Distinction of superficial and deep layering. Immunofluorescent quadruple staining was performed to reveal collagen type I expression and myofibroblast distribution within EGT. Superficial and deep layers showed positively labelled collagen type I fibres as part of the ECM (a, a‘). The fibre formations were mostly parallel to both each other and to the blood vessels, which grew perpendicular to the wound surface. Here, image acquisition was performed using the same settings, an increased amount of fluorescence was observed in the deep fibrotic layer, giving the impression of more densely packed tissue (a‘). Both layers showed positive labelling for vimentin, but there were visibly more positively stained cells in the deeper layer (b, b‘). α-SMA was also detected in both layers, but the superficial layer labelling appeared less intense (c, c‘). The nuclear counterstaining within the superficial region revealed a high number of cells that showed no vimentin-labelling. Dotted line indicates examples of blood vessels. All scale bars = 100 µm.

α-SMA upregulation and stress fibre formation is a hallmark of activated myofibroblasts. In EGT, the superficial layer was comprised of interspersed multifocally arranged α-SMA^+^ cells (Fig 4). In the deep layer, α-SMA expression was colocalized to collagen type I rich regions and vimentin^+^ cells. The cytoplasmatic signal of α-SMA gave an impression of a denser fibre arrangement than in the superficial layer, with clearly distinguishable labelling and perinuclear accumulation.

The transition from the superficial to the deep region was marked by an increased labelling for vimentin (Fig 4, S5 Fig). It was noticeable that the superficial layer included a scattered staining pattern of the anti-vimentin-antibody. The deep layer of all evaluated slides was characterized by co-immunolabelled α-SMA^+^/vimentin^+^ myofibroblasts.

### PDGFR^+^ immunofluorescent signal in endothelial and mesenchymal cells in both wound layers

**Fig 5:**
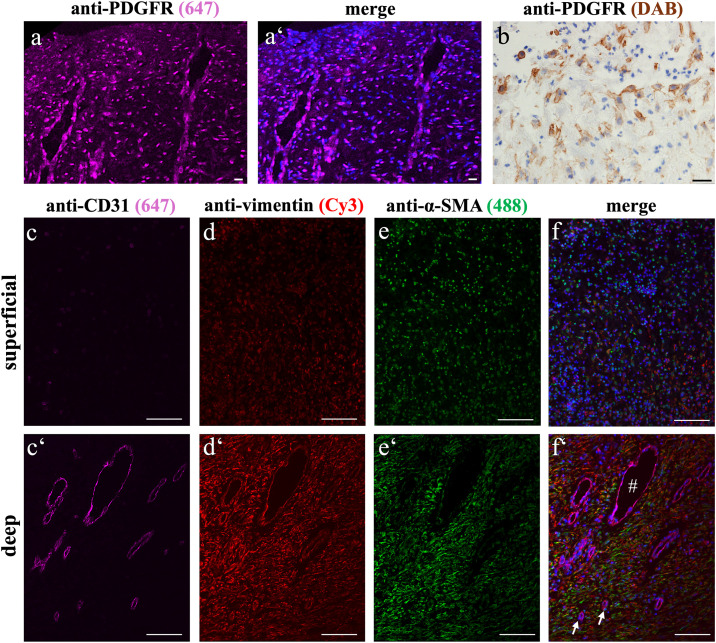
Endothelial and mesenchymal cells in both layers of EGT. The immunofluorescent staining for PDGFR (a, a’) showed positive labelling in all areas of the EGT. Endothelial as well as mesenchymal cells stained positively and showed a homogenous distribution pattern within the tissue. As the immunofluorescent labelling of PDGFR did not lead to consistently reliable results in all samples, additional immunohistochemistry was performed **(b)**, which showed a clear membranous accumulation of PDGFR expression. All scale bars = 20 µm.

Scattered PDGFR^+^ cells were observed in EGT (Fig 5). Immunostaining was observed mainly in the cytoplasmic region within the endothelial cells and pericytes. Within the tissue, occasional labelling with perinuclear accumulation was seen. The immunofluorescent staining for PDGFR could not be reliably reproduced in all samples, whereas an alternative immunohistochemistry using DAB-labelling, was consistently reproducible. The antibody showed clear membranous staining as well as scattered weak cytoplasmic stain.

CD31^+^ cells were predominantly observed in the deep regions of EGT (c, c‘). In this area, the endothelial layer of blood vessels was labelled positively for CD31 as well as vimentin (d‘), confirming their identification as endothelial cells. Less vimentin labelling was observed in the superficial layer (d). α-SMA was stained in both the deep and superficial layers (e, e‘), with higher accumulation observed in the deep layer. # = venous vessel; * = arterial vessels; All scale bars = 100 µm.

### CD31 and α-SMA labelling: Endothelial cell hypertrophy, vascular orientation and occlusion

CD31 showed clear positive labelling of the endothelial lining of blood vessels in the EGT (Fig 5). For most endothelial cells, the cell membrane was labelled. A small number of cells showed intracellular signalling. The blood vessels developed perpendicular to the wound surface and showed a parallel arrangement to one another; this was the case for both control granulation tissue and EGT. Regarding the transition of the superficial to the deep layer, it was characterized by the observation of blood vessels. Interestingly, there were several vessels observed that were not perpendicularly orientated to the wound surface (S5 Fig).

**Fig 6:**
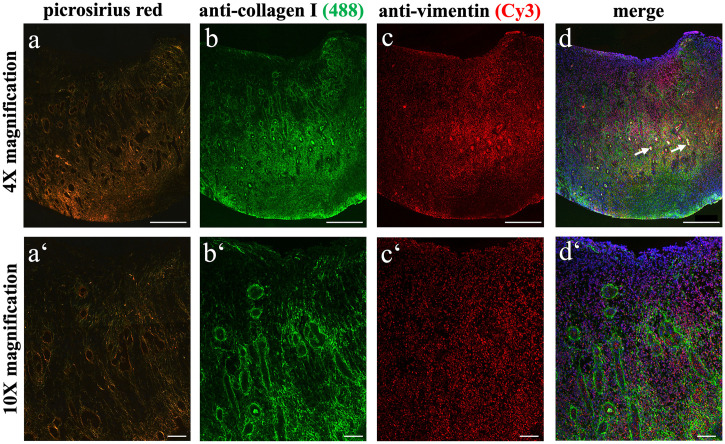
Picrosirius red and immunofluorescent collagen I staining show comparable results. Picrosirius red staining (a, a‘), previously performed to assess collagen distribution, was here directly compared to immunofluorescent staining for collagen type I (b, b‘). Both methods consistently showed more intense staining – indicating a higher fibre density – in the deeper regions of the wound bed (a“, b“). Notably, the picrosirius red stain revealed numerous red-orange fibres in the deep region (a“). Both techniques exhibited the netlike structure of the ECM between the blood vessels and also the parallel fibre alignment compared to the vessels. In contrast to picrosirius red, collagen type I also exhibited false-positive staining of erythrocytes within a number of blood vessels. Several occluded blood vessels were visible (arrows). Vimentin labelling was positive in both the superficial and deep layers (c, c‘, c“). All scale bars at 4X magnification = 500 µm; all scale bars at 10X magnification = 100 µm.

All of the blood vessels displayed a luminal CD31^+^ endothelium, followed by an α-SMA^+^ positive layer, building the tunica intima as well as a layer of vimentin-labelled mesenchymal cells. Numerous occluded blood vessels and endothelial cell hypertrophy were observed in EGT sections (Fig 6).

## Discussion

The aim of this study was to investigate the ECM composition of EGT, understand collagen type distribution within EGT tissue, link collagen deposits to the mesenchymal cells producing them, namely myofibroblasts, and to examine the associated blood vessels within the superficial and deep layers. In addition to understanding the histomorphology of EGT, the collagen findings and layer depths were compared to normal granulation tissue in wound healing as control.

The statistical analysis of the picrosirius red staining reflects increased collagen type III in the deeper region of the EGT samples compared to control wound tissue.

The collagen type I amount in the deep layers of EGT was significantly higher than in the superficial layer, which was also observed in the control group. The significantly higher green (collagen type III) versus red (collagen type I) ratio in both EGT layers compared to the control group implied higher collagen type III levels in EGT. This finding suggests more immature characteristics of EGT compared to control wounds. Usually, immature collagen type III is replaced with more collagen type I during maturation, yet in EGT this replacement seems to be delayed.

These results indicate that the difference between EGT and non-EGT wounds cannot be explained by differing total collagen amounts, but via differences in collagen maturation.

Mechanisms underlying this impaired collagen remodelling should consider the role of transforming growth factor beta (TGF-β), particularly TGF-β1. TGF-β1 stimulates myofibroblast activation, thereby increasing collagen production [29,30]. In horses, TGF-β1 levels in distal limb wounds remain significantly higher compared to body wounds, as shown in an in vivo study conducted on six horses, and this has been discussed as a contributing factor to hypergranulation [31]. Another important factor to drive EGT formation is persistent infection. Bacteria commonly associated with chronic wounds, such as *Pseudomonas aeruginosa* and *Staphylococcus aureus*, can form biofilms that interferes with TGF-β1 signaling. Interestingly, surgically created wounds on horse limbs that were induced to develop EGT by bandaging, showed significant higher rates of biofilm formation compared to unbandaged wounds [32,33].

Notably, ponies rarely develop EGT, so studies have previously investigated the differences in wound healing in distal limbs with a special focus on the comparison of the inflammatory response of horses versus ponies [33–35]. The present study supports these findings that the weak and prolonged inflammatory phase in horses results in recurrent inflammation and proliferation, thereby creating a vicious cycle that hinders progression to the remodelling phase, as previously described [15].

Wilmink et al. performed in vitro contraction capacity studies of primary isolated fibroblasts originating from limbs and buttocks [35,36], highlighting that fibroblasts and myofibroblasts act differently in vitro. Furthermore, they demonstrated that contraction capacity did not depend on fibroblast origin but was more likely dependent on the surrounding ECM and activation state the cell itself. As EGT contractibility is reduced, the activation and role of myofibroblasts in situ is of further interest. Our positive staining for α-SMA indicates the completed activation of the myofibroblast in EGT and therefore reflects a high contractibility potential. In general, after tissue injury and under increasing mechanical stress, the first step is to activate quiescent, vimentin^+^ fibrocytes towards protomyofibroblasts. Their actin cytoskeleton reorganizes leading to the development of stress fibres. The final stage of activation involves myofibroblasts, which have the highest contraction capability [37]. The reduced vimentin expression and decreased collagen type I amount, combined with the presence of α-SMA in the superficial region compared to the deep region is an interesting feature of EGT observed in the present study. As it is known that during cell division, downregulation of vimentin occurs and nestin (intermediate filament class VI, neuroepithelial stem cell protein) is required for cytokinesis [38], we strived to detect nestin immunofluorescently as well, but regrettably, this approach was unsuccessful. Together with the PDGFR^+^ labelling also observed in the superficial layer, we assume that the tissue being reverted into partly acute inflammation, remains in a highly undifferentiated state, thereby impeding regeneration.

In addition to PDGFR, we completed the picture by labelling CD31 for blood vessel detection in our EGT samples. The previously described endothelial cell hypertrophy [20,39], was also observed here. This could be explained by local hypoxic conditions which promotes upregulation of collagen synthesis [24,25]. Even though the presence of PDGFR^+^ cells in both regions of EGT indicates the upregulation of angiogenesis to promote wound healing, it is hypothesized that the pathway is disrupted in the superficial layer, which is suggestive of impeded neoangiogenesis.

Collagen detection, type distinction and quantitative estimation with picrosirius red has been performed previously in species such as sheep and rats [40,41], and represents an affordable and commonly used histological stain for investigating the ECM. Nevertheless, multiple studies have discussed the reliability of collagen distinction with picrosirius red. The resulting photomicrographs can change depending on i) fibre organization, ii) fibre orientation and iii) sample orientation on the microscopical stage [41–43]. Therefore, it is of crucial importance to control for this by including fixed exposure times, white balancing and scanning in a dark environment. Additionally, immunofluorescence staining for collagen types I and III were performed to further evaluate and compare the picrosirius red findings in EGT. Both staining methods performed on the same samples showed a comparable signal for collagen type I in EGT, they both showed an increased positive labelling in the deep layer. Anti-collagen type III antibody staining displayed the cobweb like fibre architecture in both layers of EGT, which was also seen in the picrosirius red staining (Fig 6).

There are some limitations to the present study. One is the wide age range of the horses included, which reflects the reliance on samples from equine clinics (S2 Table). Although EGT is more commonly observed in adult horses, it can also affect foals and yearlings [44]. To ensure a representative sample, all clinically confirmed cases were included regardless of age. However, age-related differences in immune function, such as the reduced phagocytic and killing capacity of neutrophils and altered cytokine expression observed in foals, may influence the inflammatory and reparative responses central to EGT development [45]. The number of control wounds (n = 6) was limited, however it is also a strength that there were control animals of the same species (previous studies have lacked same species controls [14]). For ethical reasons, this study relied on archived FFPE samples of punch biopsies from a previous study with experimentally induced wounds, limiting the number of control animals and samples. This was also the reason why the comparison of EGT wounds to experimentally induced wounds was limited to the picrosirius red analysis. Finding cross-reactive antibodies for horse tissues is a further limitation which equine studies frequently encounter. Here, cross-reactive antibodies for α-SMA, vimentin, collagen type I and III, CD31 and PDGFR were used. Anti-collagen type III and anti-PDGFR did not deliver reliable results using IF, which could be resolved by the means of IHC.

In conclusion, the findings of collagen deposition, location and vasculature provide valuable understanding of EGT and its mechanisms of action in horses. Even though the total amount of collagen in EGT compared to control wounds is similar, the composition differs. With significantly higher amounts of collagen type III, EGT is reminiscent of a more immature granulation tissue.

Myofibroblasts and immune cells are pivotal components in the pathogenesis of EGT. With the insights provided here, along with further investigations on EGT, we hope combined efforts will lead to therapeutic options so conducive to regeneration that EGT can one day be prevented.

## Supporting information

S1 Table Materials used for staining. (TIFF)

S2 Table Information on EGT horses. (TIFF)

S1 Fig Double staining of equine skin sections shows antibody specificity. For all antibodies used, a control double staining on equine FFPE skin sections was performed using the same protocol as for EGT sections. Anti-α-SMA shows clear labelling of the Mm. arrectores pilorum (a, a’) and CD31 expression was seen specifically in endothelial cells (b, b’). Both anti-collagen-antibodies label ECM components with a cobweb-like structure (c, c’, d, d’), whereas anti-collagen type III antibody also labels the sebaceous glands. Signal for PDGFR can be addressed to mainly spindle shaped cells, with membranous staining visible in the cell processes (e, e’). Vimentin-labelling is detected in the dermis showing cytoplasmic staining of mesenchymal cells like fibroblasts (f, f’). All scale bars = 25 µm.
(TIFF)

S2 Fig DAB-labelling for collagen type III and PDGFR as control on equine skin sections. As the IF staining was not reliable for collagen type III and PDGFR, additional IHC staining was performed, and validated on equine skin sections. Collagen type III is mainly labelled in the ECM (a, a’). Sebaceous glands show strong signal as seen in the IF; a false-positive DAB labelling of the epidermal layer can be recognized. For anti-PDGFR-antibody, the staining can be addressed to cells with long-shaped cellular processes, which are mostly arranged around the hair follicles (b, b’). All scale bars = 25 µm.
(TIFF)

S3 Fig Comparison of superficial layer thickness of control wounds and EGT using picrosirius red staining. Histological evaluation of granulation tissue shows a zonation of wound bed with a cell-rich superficial, and a fibrotic deep layer. Measurements of picrosirius red stained sections reveals, that the superficial layer of EGT is significantly thinner than the superficial layer of the control wounds.
(TIFF)

S4 Fig Immunohistochemical staining for collagen type III in EGT. DAB staining for collagen type III shows positive labelling in both layers of EGT (a). Superficially (b), the staining is observed to be less intensive than in the deeper layer (b‘). Endothelial cells do not show staining whereas myofibroblasts have intracellular labelling. Additionally, a cobweb like fibre network is observed. Dotted line indicates exemplary blood vessel. All scale bars = 100 µm.
(TIFF)

S5 Fig Immunofluorescent triple staining on EGT show blood vessel abnormalities. Next to the appearance of perpendicular grown blood vessels (a, a‘), the transition of the superficial to the deep layer in EGT is characterized by an observed increase of vimentin expression in the deeper layer (b, b‘). Arterial and venous vessels are observed (c‘), both of which exhibit endothelial cell hypertrophy (c‘). Aberrant vessel formation (*) is present in the tissue (c‘). Red arrowhead = arterial vessel; blue arrowhead = venous vessel; * = aberrant blood vessel; # = endothelial cell hypertrophy. All scale bars = 100 µm.
(TIFF)
