# Correction: Collagen composition in equine exuberant granulation tissue reflects tissue immaturity

**DOI:** 10.1371/journal.pone.0344466

**Published:** 2026-03-06

**Authors:** Lena Partusch, Catrin Sian Rutland, Ann Martens, Charis Du Cheyne, Ward De Spiegelaere, Jule Kristin Michler

The following information is missing from the Funding statement: The publication costs were covered by the Open Access Funds of the University of Veterinary Medicine Vienna.
