# Supplementary figures and images for: Collagen composition in equine exuberant granulation tissue reflects tissue immaturity
Source: PLoS One. 2025 Nov 6;20(11):e0335179. doi: 10.1371/journal.pone.0335179 (PMC12591489; doi:10.1371/journal.pone.0335179)

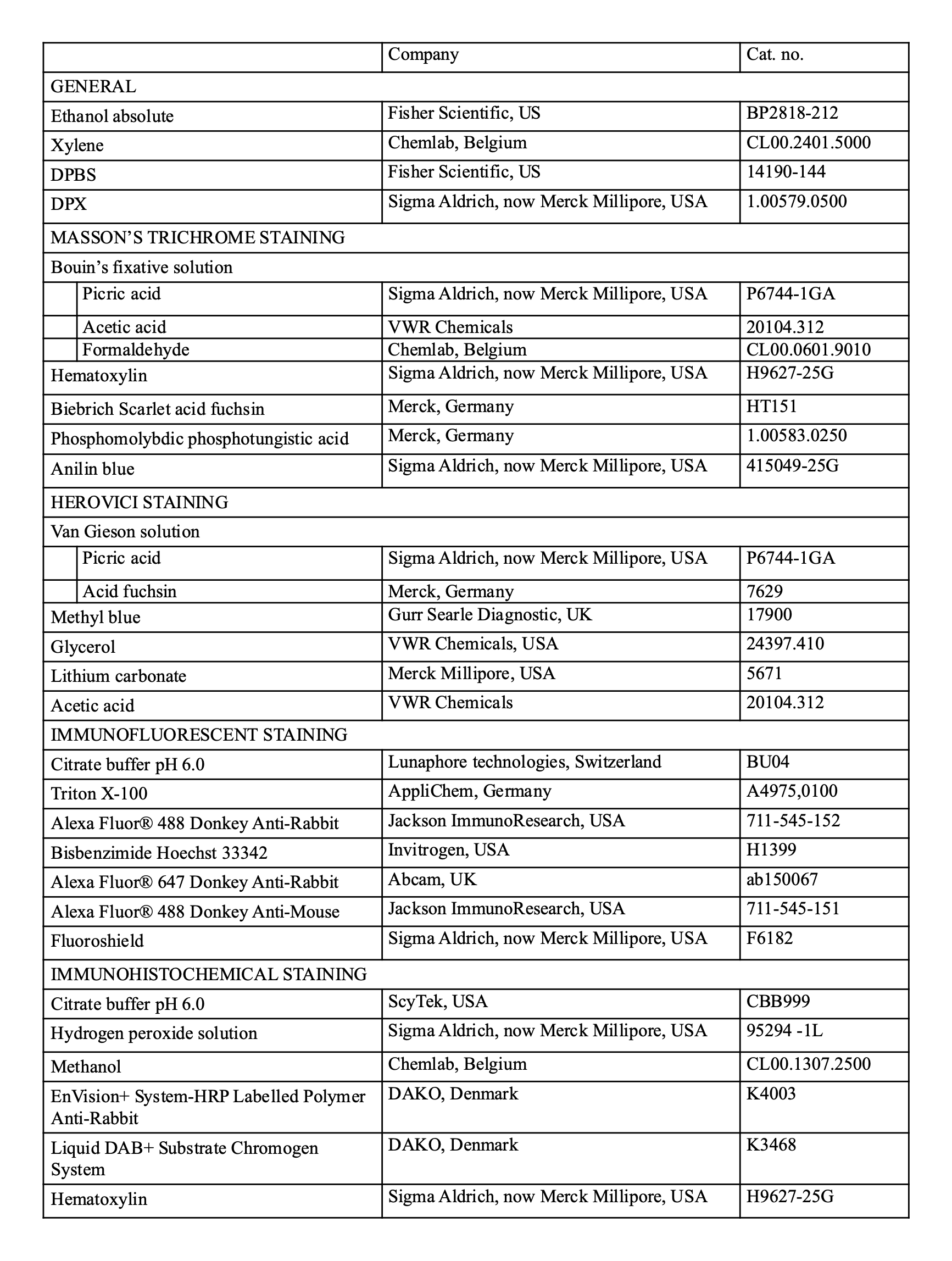

Supplement: S1 Table — (TIFF) [file pone.0335179.s001.tiff]

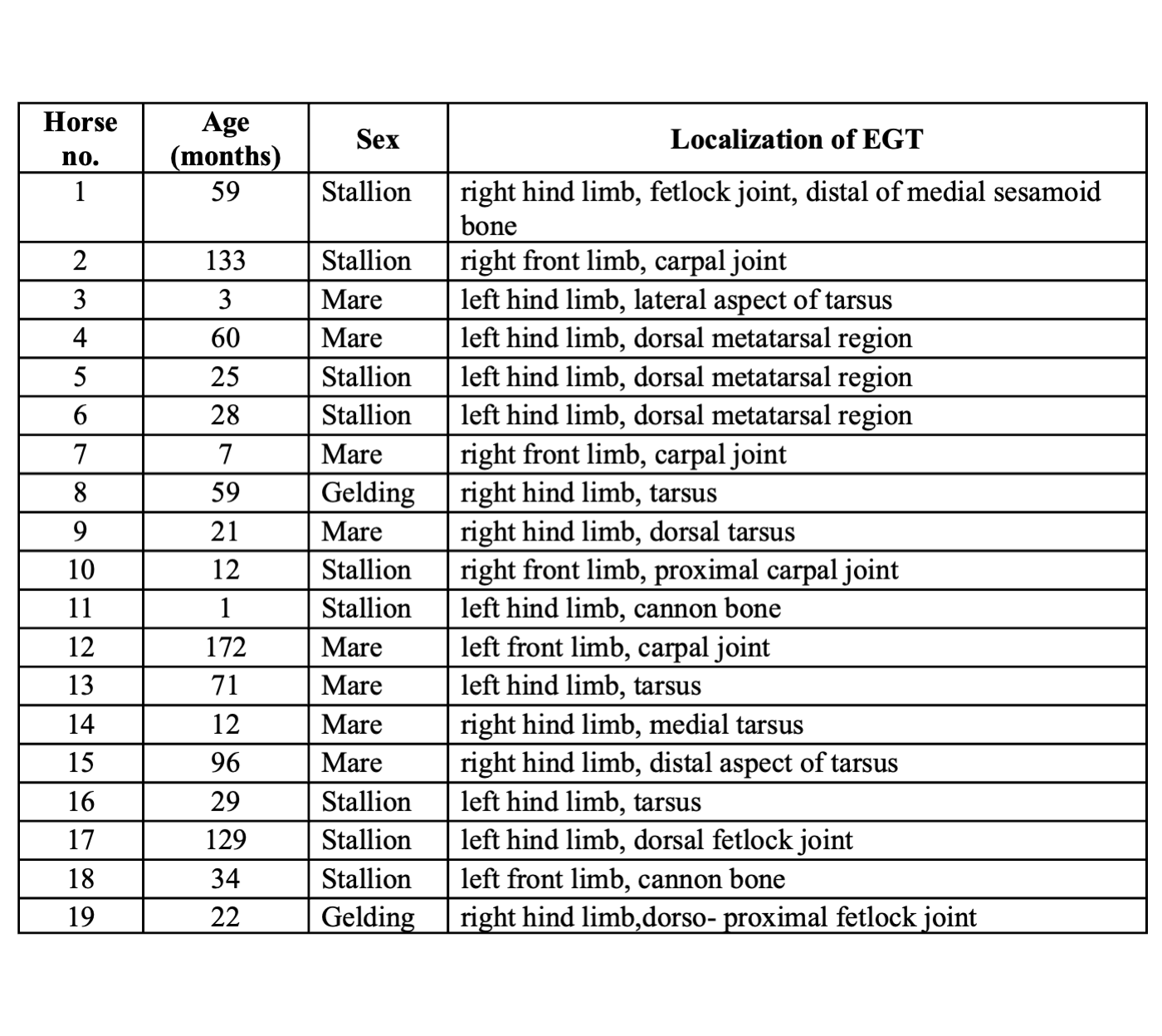

Supplement: S2 Table — (TIFF) [file pone.0335179.s002.tiff]

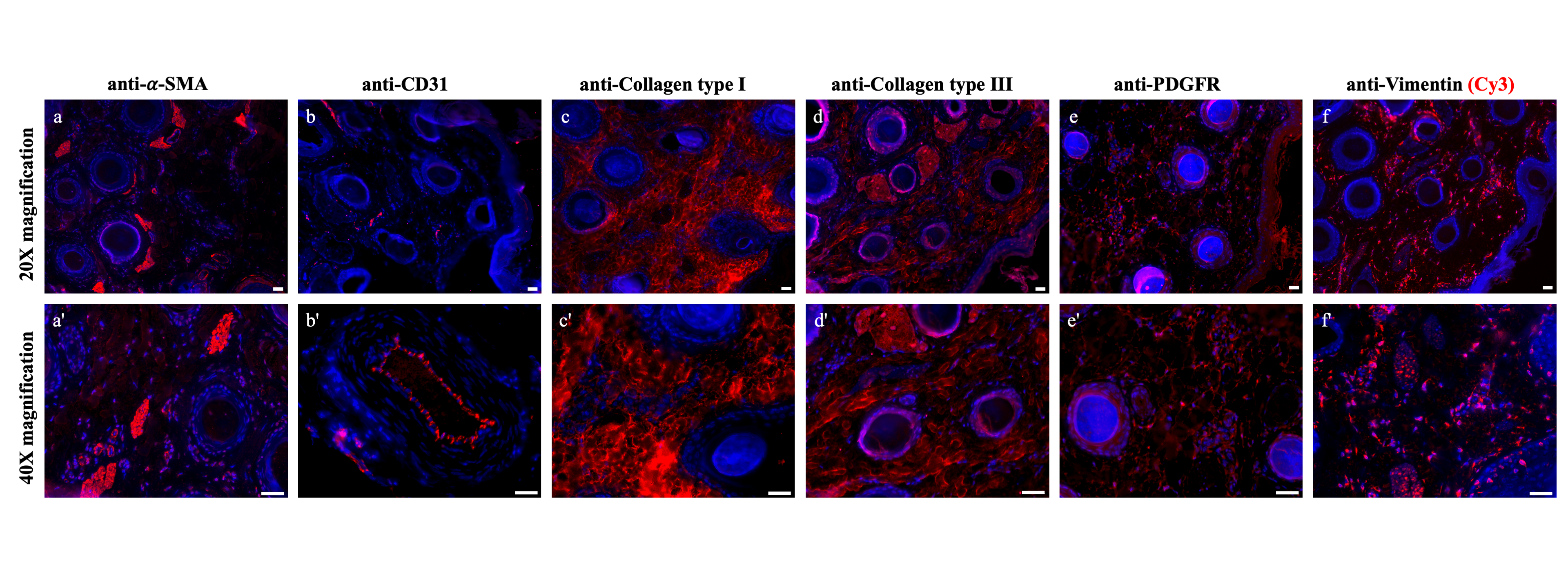

Supplement: S1 Fig — For all antibodies used, a control double staining on equine FFPE skin sections was performed using the same protocol as for EGT sections. Anti-α-SMA shows clear labelling of the Mm. arrectores pilorum (a, a’) and CD31 expression was seen specifically in endothelial cells (b, b’). Both anti-collagen-antibodies label ECM components with a cobweb-like structure (c, c’, d, d’), whereas anti-collagen type III antibody also labels the sebaceous glands. Signal for PDGFR can be addressed to mainly spindle shaped cells, with membranous staining visible in the cell processes (e, e’). Vimentin-labelling is detected in the dermis showing cytoplasmic staining of mesenchymal cells like fibroblasts (f, f’). All scale bars = 25 µm. (TIFF) [file pone.0335179.s003.tiff]

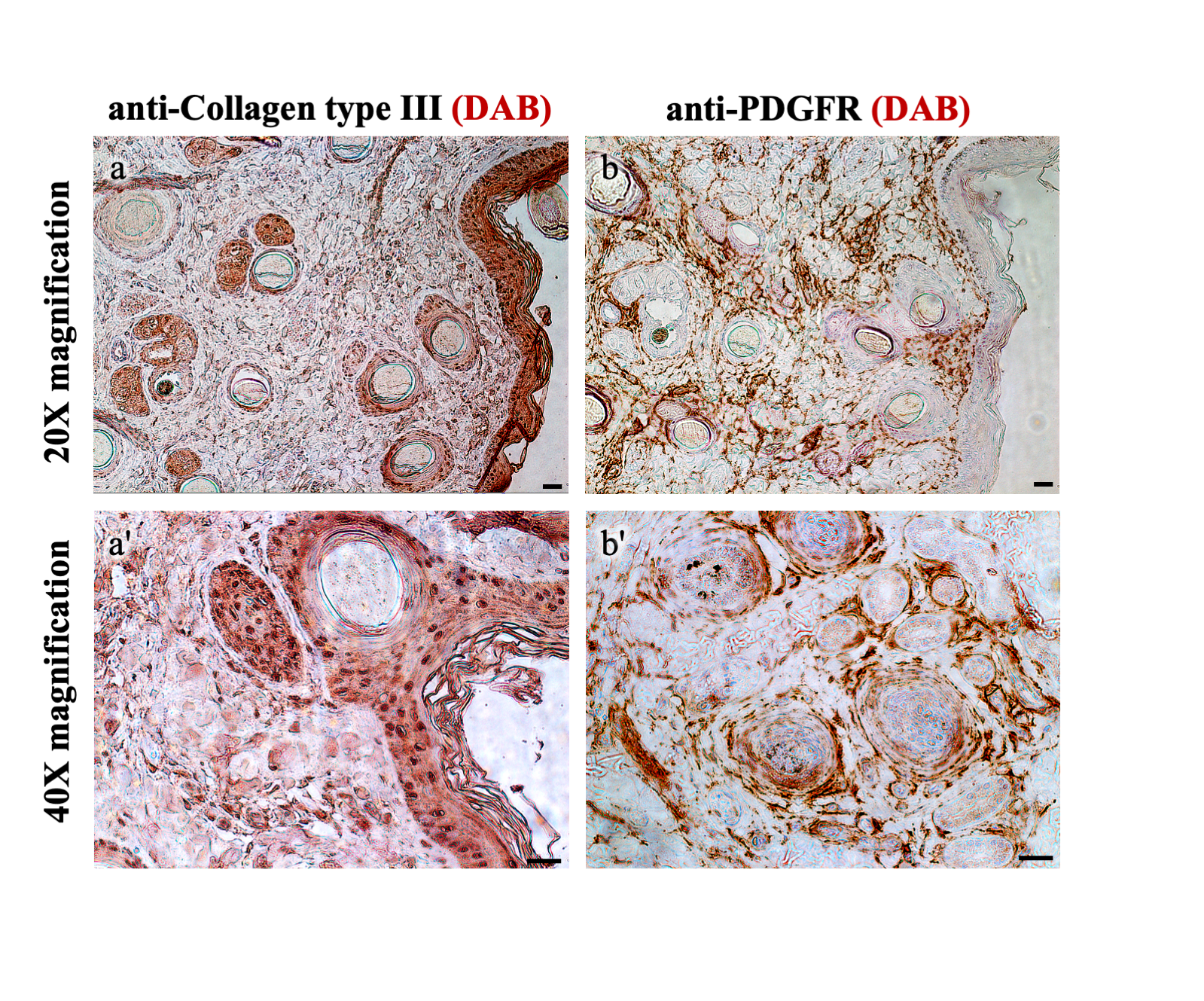

Supplement: S2 Fig — As the IF staining was not reliable for collagen type III and PDGFR, additional IHC staining was performed, and validated on equine skin sections. Collagen type III is mainly labelled in the ECM (a, a’). Sebaceous glands show strong signal as seen in the IF; a false-positive DAB labelling of the epidermal layer can be recognized. For anti-PDGFR-antibody, the staining can be addressed to cells with long-shaped cellular processes, which are mostly arranged around the hair follicles (b, b’). All scale bars = 25 µm. (TIFF) [file pone.0335179.s004.tiff]

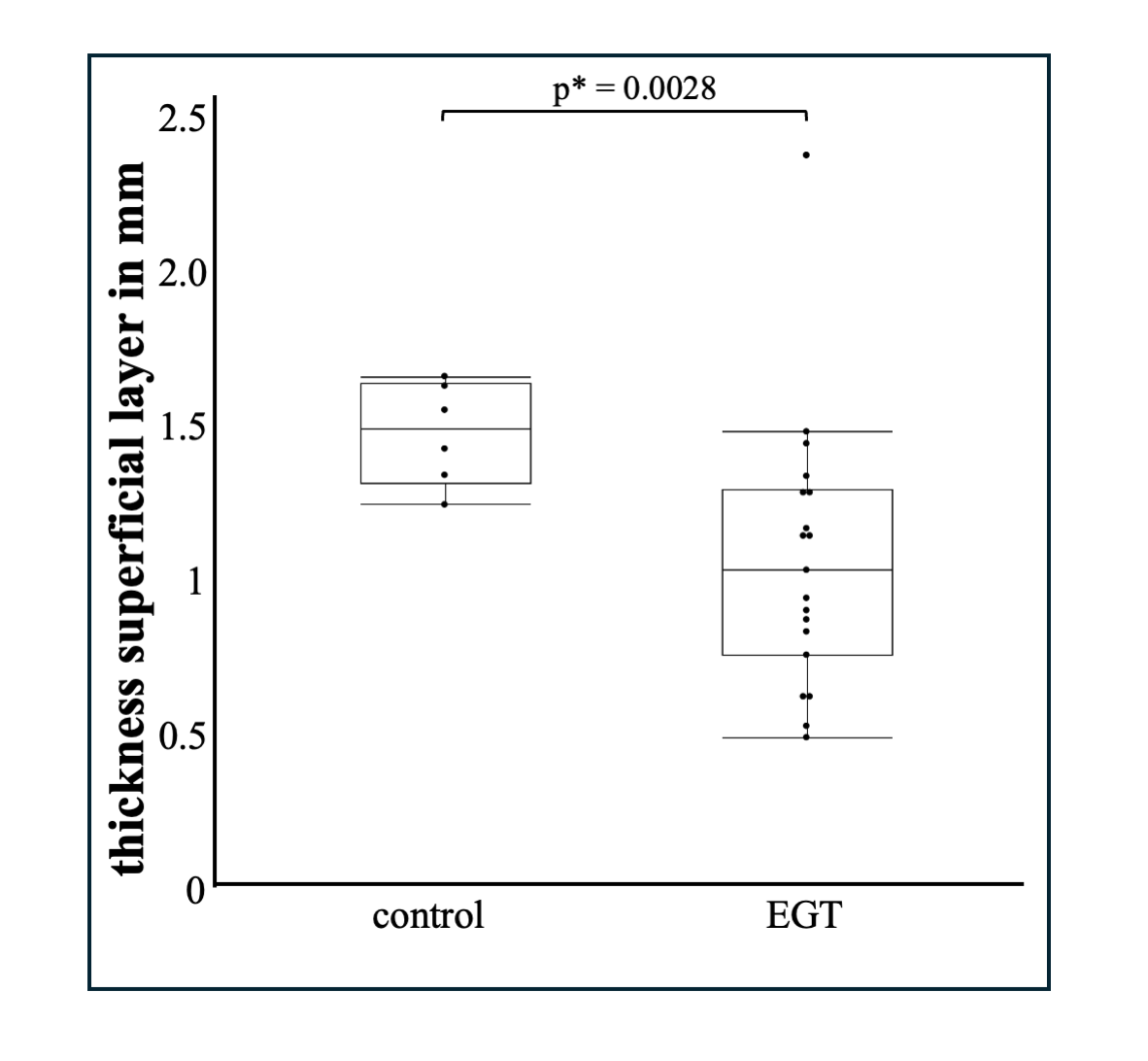

Supplement: S3 Fig — Histological evaluation of granulation tissue shows a zonation of wound bed with a cell-rich superficial, and a fibrotic deep layer. Measurements of picrosirius red stained sections reveals, that the superficial layer of EGT is significantly thinner than the superficial layer of the control wounds. (TIFF) [file pone.0335179.s005.tiff]

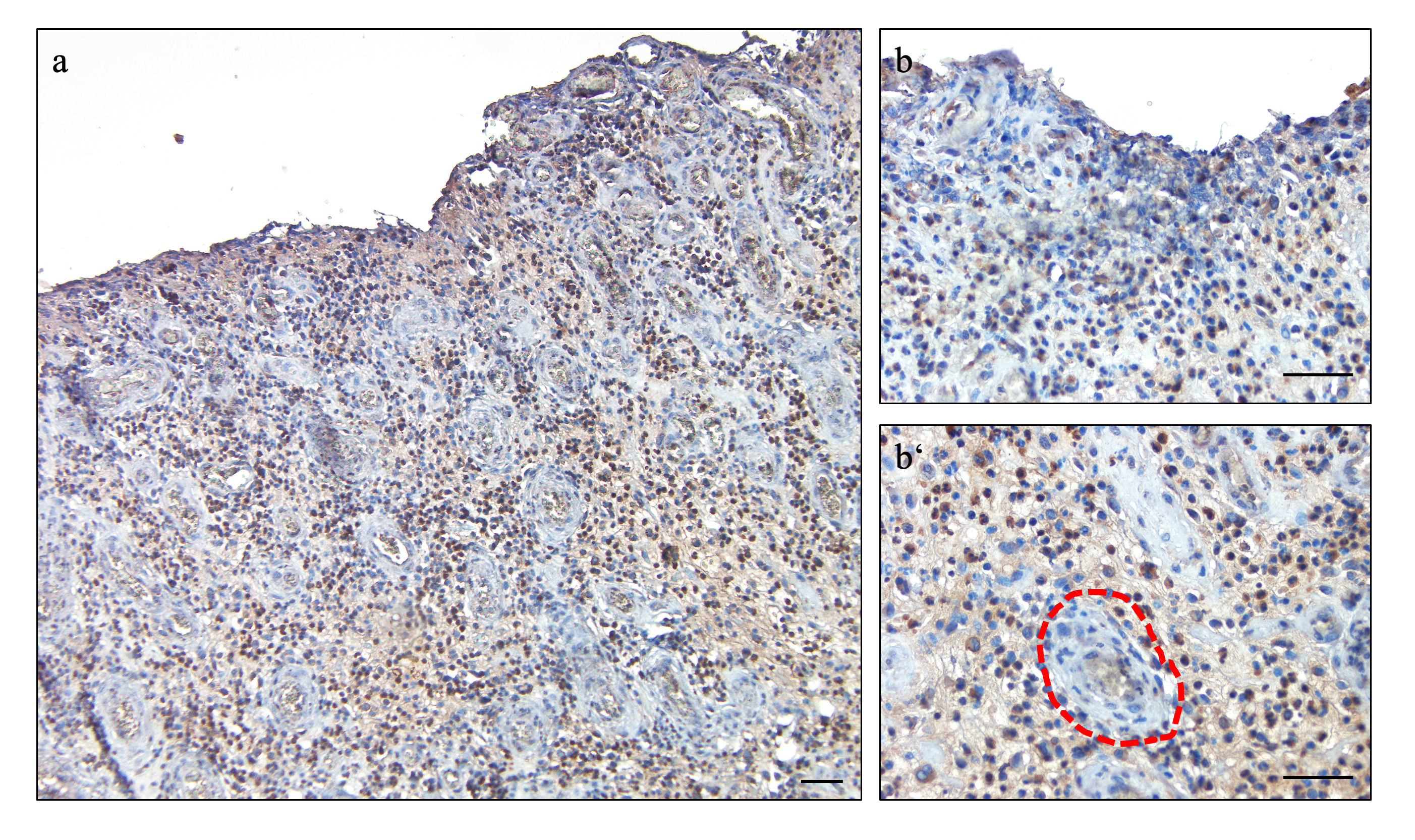

Supplement: S4 Fig — DAB staining for collagen type III shows positive labelling in both layers of EGT (a). Superficially (b), the staining is observed to be less intensive than in the deeper layer (b‘). Endothelial cells do not show staining whereas myofibroblasts have intracellular labelling. Additionally, a cobweb like fibre network is observed. Dotted line indicates exemplary blood vessel. All scale bars = 100 µm. (TIFF) [file pone.0335179.s006.tiff]

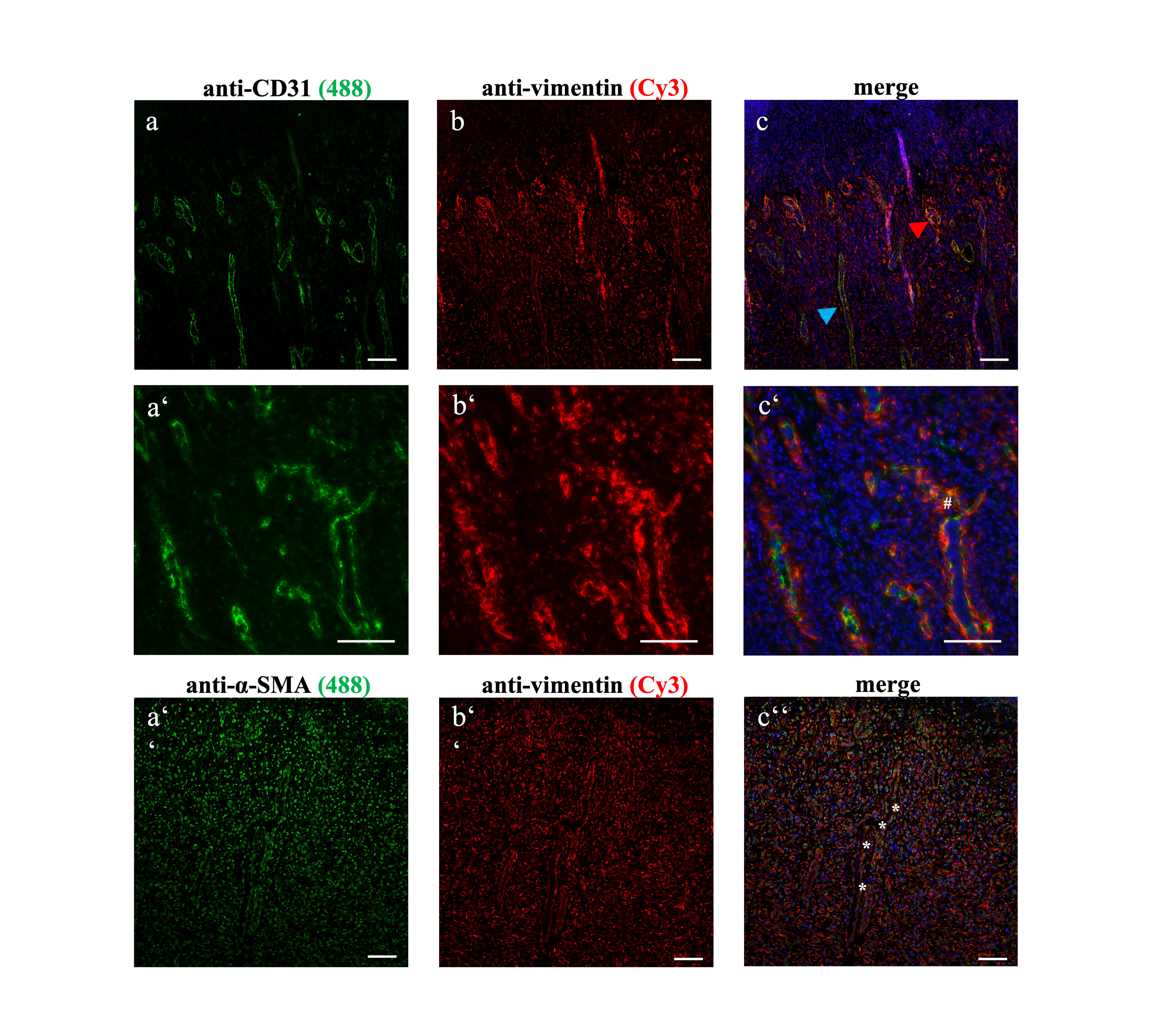

Supplement: S5 Fig — Next to the appearance of perpendicular grown blood vessels (a, a‘), the transition of the superficial to the deep layer in EGT is characterized by an observed increase of vimentin expression in the deeper layer (b, b‘). Arterial and venous vessels are observed (c‘), both of which exhibit endothelial cell hypertrophy (c‘). Aberrant vessel formation (*) is present in the tissue (c‘). Red arrowhead = arterial vessel; blue arrowhead = venous vessel; * = aberrant blood vessel; # = endothelial cell hypertrophy. All scale bars = 100 µm. (TIFF) [file pone.0335179.s007.tiff]
